# Using a priority setting exercise to identify priorities for guidelines on newborn and child health in South Africa, Malawi, and Nigeria

**DOI:** 10.1186/s12961-024-01133-7

**Published:** 2024-04-16

**Authors:** Solange Durão, Emmanuel Effa, Nyanyiwe Mbeye, Mashudu Mthethwa, Michael McCaul, Celeste Naude, Amanda Brand, Ntombifuthi Blose, Denny Mabetha, Moriam Chibuzor, Dachi Arikpo, Roselyn Chipojola, Gertrude Kunje, Per Olav Vandvik, Ekpereonne Esu, Simon Lewin, Tamara Kredo

**Affiliations:** 1 Health Systems Research Unit, South African Medical Research Council, Cape Town, South Africa; 2 Cochrane Nigeria, Institute of Tropical Diseases Research and Prevention, University of Calabar Teaching Hospital, Calabar, Nigeria; 3 Evidence Informed Decision-Making Centre, Department of Community and Environmental Health, School of Global and Public Health, Kamuzu University of Health Sciences, Blantyre, Malawi; 4 Centre for Evidence-Based Health Care, Division of Epidemiology and Biostatistics, Department of Global Health, Faculty of Medicine and Health Sciences, Stellenbosch University, Cape Town, South Africa; 5 MAGIC Evidence Ecosystem Foundation, Oslo, Norway; 6 Department of Medicine, Lovisenberg Diaconal Trust, Oslo, Norway; 7 Department of Health Sciences Alesund, Norwegian University of Science and Technology, Trondheim, Norway

**Keywords:** Clinical practice guidelines, Newborn, Infant and child health, Priority setting, Sub-Saharan Africa, Health policy

## Abstract

**Background:**

Sub-Saharan Africa is the region with the highest under-five mortality rate globally. Child healthcare decisions should be based on rigorously developed evidence-informed guidelines. The Global Evidence, Local Adaptation (GELA) project is enhancing capacity to use global research to develop locally relevant guidelines for newborn and child health in South Africa (SA), Malawi, and Nigeria. The first step in this process was to identify national priorities for newborn and child health guideline development, and this paper describes our approach.

**Methods:**

We followed a good practice method for priority setting, including stakeholder engagement, online priority setting surveys and consensus meetings, conducted separately in South Africa, Malawi and Nigeria. We established national Steering Groups (SG), comprising 10–13 members representing government, academia, and other stakeholders, identified through existing contacts and references, who helped prioritise initial topics identified by research teams and oversaw the process. Various stakeholders were consulted via online surveys to rate the importance of topics, with results informing consensus meetings with SGs where final priority topics were agreed.

**Results:**

Based on survey results, nine, 10 and 11 topics were identified in SA, Malawi, and Nigeria respectively, which informed consensus meetings. Through voting and discussion within meetings, and further engagement after the meetings, the top three priority topics were identified in each country. In SA, the topics concerned anemia prevention in infants and young children and post-discharge support for caregivers of preterm and LBW babies. In Malawi, they focused on enteral nutrition in critically ill children, diagnosis of childhood cancers in the community, and caring for neonates. In Nigeria, the topics focused on identifying pre-eclampsia in the community, hand hygiene compliance to prevent infections, and enteral nutrition for LBW and preterm infants.

**Conclusions:**

Through dynamic and iterative stakeholder engagement, we identified three priority topics for guideline development on newborn and child health in SA, Malawi and Nigeria. Topics were specific to contexts, with no overlap, which highlights the importance of contextualised priority setting as well as of the relationships with key decisionmakers who help define the priorities.

**Supplementary Information:**

The online version contains supplementary material available at 10.1186/s12961-024-01133-7.

## Background

Globally, more than half of all deaths in children and youth in 2019 were among children under 5 years [1]. It is estimated that there are 5.2 million deaths among under-fives each year, with Sub-Saharan Africa (SSA) having the highest mortality rate [2]. Most countries in SSA are not on track to meet maternal and child health targets set by Sustainable Development Goal 3 to ‘ensure healthy lives and promote wellbeing’, specifically the target of 25 or fewer deaths per 1000 live births [1]. As of December 2021, under-five mortality rates were reported as 113.8, 38.6 and 32.2 deaths per 1000 live births for Nigeria, Malawi and South Africa respectively [3]. Factors accounting for regional disparities in child mortality rates include poverty, socioeconomic inequities, poor health systems, and poor nutrition, with disease outbreaks adding substantially to the burden [4].

Addressing these issues requires an evidence-informed approach to ensure that scarce resources are used effectively and efficiently, avoid harm, maximise benefits, and improve healthcare delivery and outcomes [5–7]. Evidence-informed practices have been growing in SSA [5], and include the use of recommendations from clinical practice guidelines that are intended to optimise patient care or public health practice [8]. Guidelines bridge the gap between research evidence and practice and are recognised as important quality-improvement tools that aim to standardise care, inform funding decisions, and improve access to care, amongst others.

The development of evidence-informed, trustworthy guidelines from scratch—also known as de novo guideline development—is a resource-intensive and time-consuming process [9]. However, guideline developers can adopt or adapt existing recommendations from guidelines developed in other settings, to make the process more efficient while maintaining transparency and minimising waste and duplication [10–13]. For example, the World Health Organisation (WHO) produces high-quality global-level guidelines, which may be adopted and implemented in a member country or, alternatively, adapted for that context. Guidelines can be adopted when there is no need to change the recommendation, the evidence base, or how it is implemented in a local setting while considering factors such as cost, workforce, health systems, management options and access to care [10]. They can also be adapted when there is a need to modify a guideline(s) or recommendation(s) produced in one cultural and organisational setting for application in a different context [10]. However, adaptation of such guidelines to national contexts is often not well described [14]. An evaluation of experiences of guideline adaptation across WHO regions found that adaptation is understood and implemented in a variety of ways across countries [15].

Furthermore, reporting of guidelines in the African context is usually below global standards, specifically regarding their rigor of development. Kredo and colleagues reviewed Southern African Development Community (SADC) guidelines on five specific diseases published between 2003 and 2010 and besides poor reporting they found that guidelines needed broader stakeholder involvement and greater transparency [16]. Scoping reviews of newborn and child health guidelines in South Africa, Malawi and Nigeria published between 2017 and 2022 and of pre-hospital clinical guidance in sub-Saharan Africa found that the methods and reporting of the identified guidelines do not adhere to global standards [17, 18].

In terms of priority setting processes for guideline development in African settings, little information is available. A scoping review of studies describing prioritization exercises published up to July 2019 did not identify any studies from African countries, with most studies being from Europe [19]. There is thus room for strengthening and supporting guideline development and adaptation in SSA, including the initial priority setting for those guidelines.

The Global Evidence, Local Adaptation (GELA) project focuses on addressing some of these gaps, including improving guideline development processes in SSA. The project aims to maximise the impact of research on poverty-related diseases by enhancing decision makers’ and researchers' capacity to use global research, including existing high-quality global guidelines, to develop locally relevant guidelines for newborn and child health in three sub-Saharan Africa countries: South Africa (SA), Malawi and Nigeria. The first step of this project was to identify priorities for newborn and child health guideline development in each country, and this paper aims to describe our approach.

### Priority setting methods

Priority setting is an important step in guideline development [9]. It enables the identification of the most important issues through an iterative, inclusive and explicit process [7, 20], and ensures efficient resource use by identifying topics for which guidelines are truly needed [19, 21].

**Fig. 1:**
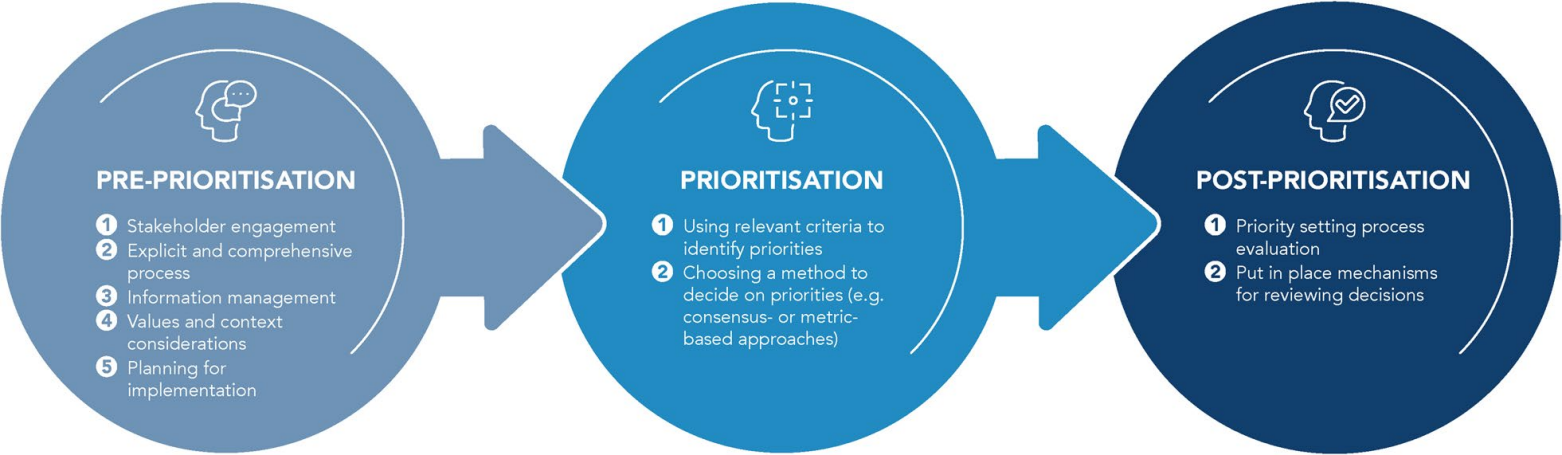
Elements of each priority setting stage (adapted from El-Harakeh 2020, Jo 2015, Sibbald 2009, Tong 2019, Viergever 2010)

A variety of methods and approaches for priority setting for research and for guidelines have been used in the literature but there is no existing gold standard method for priority setting [19, 22]. Researchers have analysed priority setting exercises and proposed good practice principles that can be followed during such exercises [22, 23]. These principles, or elements, of priority setting are linked to the different stages in the process: pre-prioritisation, prioritisation, and post-prioritisation stages, as described in Fig. 1 [19, 22–25]. In the pre-prioritisation stage, they include (i) involving internal and external stakeholders in the decision-making process, (ii) use of an explicit and transparent process, (iii) information management, (iv) consideration of values and context in which the priorities are being set, including those of stakeholders, staff and patients, and (v) planning for implementation, i.e. planning for translation of the priorities into practice. In the prioritization stage they include (i) using relevant criteria to identify priorities and (ii) choosing a method to decide on priorities, which could be consensus-based, such as the 3D Combined Approach Matrix (CAM), or metric-based approaches, such as the Child Health and Nutrition Research Initiative (CHNRI) approach [26]. In the post-prioritisation phase, they include (i) an evaluation of the priority setting process, and (sii) putting in place mechanisms for reviewing decisions.

## Methods

**Fig. 2:**
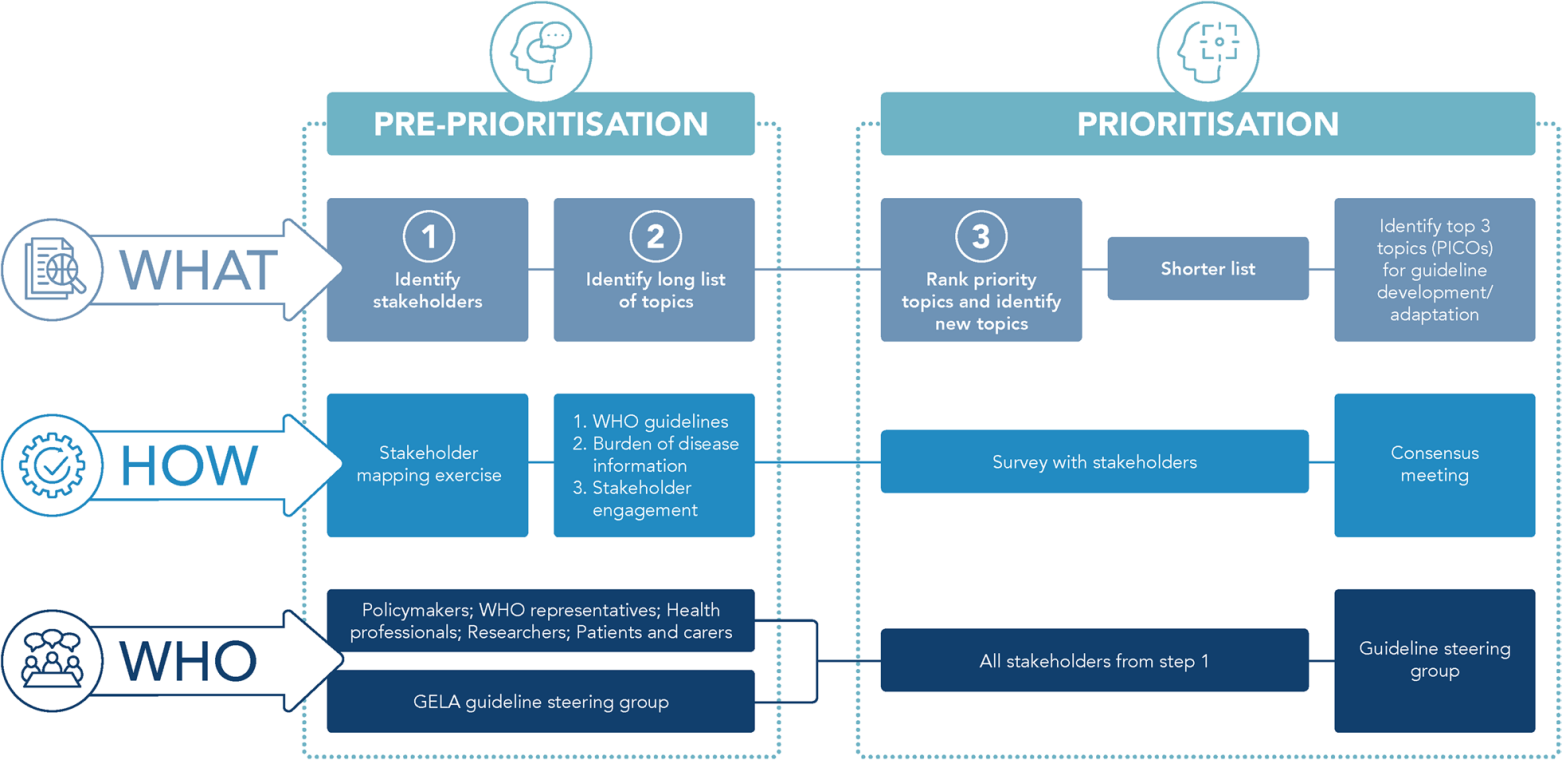
Overview of priority setting approach overview

We followed good practice priority setting method, as described above. Our approach included a pre-prioritisation stage to identify potential priority topics through stakeholder engagement and review of the literature, and a prioritisation stage for consultation and finalisation of the priority topics through online surveys and consensus meetings, using specific criteria (Fig. 2). Country teams were responsible for implementing each step in their respective countries and any differences in the process due to practical or other factors within the three countries were captured as part of the documentation of the process.

### Pre-prioritization

#### Stakeholder identification and engagement

We engaged with two different pre-specified groups. The first were the members of the Guideline steering group set up in each GELA project country. Up to 13 individuals were identified and invited to participate from the relevant national departments or ministries of health, professional associations, country-level WHO offices, and any other individuals suggested by these bodies in each country. They were identified through existing contacts of the researcher team within national departments of health responsible for guideline development and working within newborn and child health area, with whom they had worked before. These individuals then also made suggestions of other members from other stakeholder groups such as academia, non-governmental organisations, etc., who worked in the field of newborn and child health. The steering group provided initial suggestions of priority topics, made the final decisions regarding which to prioritise, and provided general oversight and technical advice on the in-country implementation of the project.

The second stakeholder group was broader and included individuals or organisations who are involved, can affect or are affected by national decisions or actions related to priority topics in the field of newborn or child health in sub-Saharan Africa [27, 28]. These included policymakers, guideline developers, health professionals, civil society representatives, patient advocacy groups as well as WHO Afro representatives, specifically those linked to potential priority topics identified. To identify them, we carried out a stakeholder mapping exercise, which included reviewing secondary data, such as existing guidance and publications and searching the websites of ministries of health, relevant professional associations, universities, NGOs and civil society groups. The national GELA Guideline Steering group members also made suggestions. This process was guided by the stakeholder power-interest matrix where those who have the most influence, and capacity to change practice for impact were prioritised and invited [29]. Participants were invited, via email, to complete a priority setting survey.

#### Identifying a long list of topics

We generated an initial long list of potential priority topics through (i) reviewing existing and planned WHO guidelines on newborn and child health, from which potential topics were extracted based on existing recommendations; (ii) consulting with the GELA guideline Steering Group; and (iii) reviewing the disease burden/technical data related to newborn and child health in each country, which was identified through targeted literature searches. WHO guidelines were used as a starting point as these are prepared following rigorous methods and are intended for implementation across the various member countries. Potential topics were organised according to the disease/condition being addressed and the type of intervention (e.g., diagnosis, prevention, treatment, or rehabilitation), and were collated into a spreadsheet or word document.

### Prioritization stage

#### Online survey

The potential topics identified in the first phase were included in online surveys with stakeholders. We first user-tested the survey among the GELA project team to ensure it was readable and understandable. We then invited all identified stakeholders, via email, to complete the survey developed using REDCap [30]. In Nigeria, the survey invitation was also circulated via WhatsApp to specific stakeholders. In the invitation emails, we also asked stakeholders to forward the email to any colleagues that may have an interest in the topic.

The landing page of the survey provided information about the study’s purpose, that it was a collaboration with the national department/ministries of health, what we were asking participants to do, and a link for more details about the GELA project, after which participants were required to provide consent before they could complete the survey. The survey asked respondents to rate the listed topics according to five criteria (Box 1) using a 6-point Likert scale (6—very critical and 1—not important at all) [22, 31]. The criteria used were identified through a survey conducted with the GELA research team in which they rated the top five criteria of 22 criteria for priority setting for guidelines identified by El Harakeh et al. [32]. An explanation for each criterion was provided in the survey. Originally, we had intended that stakeholders would rate each topic according to each criterion, but we asked instead that they consider the five criteria as a whole when rating a topic. We decided that this approach was less onerous for survey respondents and less likely to lead to poor response rates. Topics rated as being of ‘critical importance’ and ‘very critical importance’ were selected for presentation at the consensus meetings with the Steering Group. The first part of the survey also collected demographic information such as type of stakeholders, what they are primarily practicing as and for how many years, the percentage of time spent in patient care, and the type of institution they are primarily based at.

The surveys remained open for 3–4 weeks. Reminder emails were sent to those who were originally invited to participate, once in SA and Malawi, and weekly in Nigeria. As we could not track emails forwarded to others, due to the anonymity setting of the survey, it was not possible to remind those who had been invited in this manner.

Box 1. Criteria used in the priority setting process

1. Health burden—whether there is a high impact of the health problem/condition in the country as measured by financial cost, mortality, morbidity, or other indicators (e.g. QALYs, DALYs)
2. Urgency—whether there is an urgent need to address the issue or practice gap
3. Absence of guidance—whether there are no up-to-date existing guidelines addressing the specific topic and the topic would fit into existing national guideline development processes and priorities
4. Impact on health outcomes—whether a recommendation on the topic would have a beneficial impact on health outcomes in the country
5. Feasibility of intervention implementation—whether a guideline/recommendation addressing the topic would be feasible to implement in the national context (i.e. if this is through recognised guideline development bodies

#### Steering Group consensus meeting

Each country convened a meeting of their Guideline Steering Group to identify the final top three priority topics for guideline development. The number of topics per country was based on the number of recommendations that could be addressed in each country over the broader project period given the resources available. The moderator was a member of the research team and guided the meetings and discussions. This meeting was online in South Africa, and in-person in Malawi and Nigeria. We adopted a modified Nominal Group Technique [33] to achieve consensus, including five steps:

Step 1: the research team presented a summary of how the topics for the online survey were identified, which included consultation with the same steering group, as well as the results of the survey, including the summary of the topics that were rated as critically and very critically important, which needed further prioritisation.

Step 2: With the help of the moderator the group discussed each topic to ensure that all members understood them in the same way, and we elicited their thoughts on the ratings from the survey.

Step 3: The steering group members were asked to vote, anonymously, on the topics rated as critical or very critical in the survey using a Zoom poll (South Africa) or manually using post-its (Malawi). In Nigeria, the steering group decided to reach consensus through discussion to ensure full ownership of the resulting topics by every member of the group In South Africa only one round of voting was done as the members felt there was sufficient consensus after that. When voting, members ranked the importance of each topic on a six-point Likert scale against the same five criteria used in the online survey.

Step 4: The moderator summarised the results of the voting using bar charts to visualise the rating frequency distribution [34]. The variations for ratings observed were discussed among the group, including potential explanations before another round of voting, in cases where this took place.

Step 5: Consensus on the top three topics was reached. It was originally anticipated that at the end of this meeting three priority topics—formulated as questions in Population, Intervention, Comparator, and Outcomes (PICO) format—would have been identified for the next stages of the GELA project. However, the topics identified were broad, and further scoping of the literature and existing guidelines were needed to unpack and refine them. Several subsequent meetings with the Steering Groups were therefore needed in each country to present this scoping and finalise the three priority PICO questions per country.

## Data management and analysis

Data were exported from the REDCap data management software, cleaned, and analysed using R studio [35] or STATA 12 [36]. Simple descriptive statistics were used during the analysis. Median and interquartile ranges (IQRs) were used to rank the topics, which were presented graphically and tabulated in descending order from very critically important to not important at all. Frequencies and proportions were used to describe categorical data. Response rates and missing data were noted. The data were considered as missing at random.

## Results

### Pre-prioritisation

#### Stakeholder identification and engagement

The members identified and invited to join the Steering Group in each country are described in Table 1.

**Table 1:** Stakeholders represented in the Steering Groups

South Africa (*n* = 10)	Malawi (*n* = 13)	Nigeria (*n* = 13)
Ministry of Health	Ministry of Health	Department of Family Health
National Paediatric Hospital Guideline Committee members	Neonatologist	Department of Health Planning, Research and Statistics
UNICEF SA	Academic (Kamuzu University of Health Sciences)	Cochrane Nigeria Advisory Group
South African Paediatrics Association	Paediatric Surgeon	Paediatric Association of Nigeria
WHO South Africa	Nurses and Midwives Council of Malawi	Nigeria Society of Neonatal Medicine
Committee on Mortality and Morbidity in Children	Medical Council of Malawi	Society of Gynaecology and Obstetrics of Nigeria
Red Cross Children’s Hospital	Save the Children	Association of Public Health Practitioners of Nigeria
People’s Health Movement—SA	WHO Malawi	National Association of Nigerian Nurses and Midwives
Paediatrics Academic Units Committee	Paediatric and Child Health Association	National Primary Health Care Development Agency
	UNICEF	WHO Nigeria
		UNICEF Nigeria
		Save the children Nigeria

Stakeholder mapping identified a range of stakeholders for the survey, including 78 in South Africa, 31 in Malawi, and 40 in Nigeria (Table 2). In South Africa, there was greater representation from researchers/academics and health professionals; in Malawi from policymakers and researchers/academics; and in Nigeria from policymakers and professional associations, most of whom were academics.

**Table 2:** Number and types of stakeholders identified for the survey across the three countries

Stakeholder Category*	No. of Stakeholders identified		
	South Africa	Malawi	Nigeria
Policymakers/Ministries of Health	10	11	18
Health professionals	20	0	0
Professional associations	3	4	8
Civil society representatives	8	1	3
Non-Governmental Organisations/NPOs	12	3	7
Researchers/ Academics	21	10	0
UN Agencies (WHO and UNICEF country offices)	4	2	4
Total	78	31	40

*Some individual stakeholders fit into more than one stakeholder category, e.g. health professionals/professional associations/policymakers

#### Initial list of topics

In South Africa and Nigeria, the initial lists of topics identified were very long; over 65 topics, across 14 broad topic areas in South Africa, and 51 topics in Nigeria. Through engagement and input from the respective Steering Group members and other experts—via virtual meetings or email—the lists were narrowed down. The survey in South Africa included 14 topics across six conditions, in Nigeria 27 topics across 10 conditions, and in Malawi 30 topics across eight conditions (Table 3). Aside from undernutrition, which was a broad topic included in the surveys of all three countries, there was very little overlap in topics across all the countries. Pneumonia/acute respiratory infection and tuberculosis were common to Malawi and Nigeria, and schistosomiasis was common to South Africa and Malawi.

**Table 3:** Broad conditions and number of specific topics included in the surveys

Broad conditions	Number of topics included in the survey		
	South Africa	Malawi	Nigeria
Birth defects	–	–	1
Breastfeeding	–	–	–
Cerebral palsy	1	–	–
COVID-19	–	2	–
Diarrhoeal disease	–	–	3
HIV/AIDS	–	5	–
Immunization coverage	–	–	3
Malaria	–	3	–
Neonatal sepsis/Serious newborn infections	–	–	4
Newborn care	–	–	4
Newborn eye conditions	–	–	–
Obesity	2	–	–
Perinatal asphyxia	4	–	–
Pneumonia/ARIs	–	2	2
Polio	–	2	–
Prematurity	2	–	–
Pre-term birth complications	–	–	4
Schistosomiasis	1	5	–
Tuberculosis	–	7	2
Trachoma	–	–	1
Undernutrition (including stunting, SAM, MAM)	4	4	3
Total	14	30	27

AIDS: acquired immunodeficiency syndrome; ARIs: Acute Respiratory Infections; COVID-19: coronavirus disease 2019; HIV: human immunodeficiency virus; MAM: moderate acute malnutrition; SAM: severe acute malnutrition

### Prioritisation

#### Online survey

All the stakeholders described in Table 2 were invited to complete the online survey via email. In Nigeria, WhatsApp messages were also sent to representatives of professional groups (paediatricians, Obstetricians, neonatologists), who then shared on their groups. The surveys were open for approximately three to four weeks at the end of 2022: 10 October to 8 November in South Africa; 7 to 25 November in Malawi; and 10 November to 3 December in Nigeria. Whereas in South Africa and Malawi fewer people accessed the survey compared to the number of people invited (38/78 in SA, 23/61 in Malawi), in Nigeria a greater number of people accessed it (78/57). However, a similar percentage of those that accessed the survey fully completed it (66% in SA, 70% in Malawi, and 68% in Nigeria). Some respondents completed the first part of the survey, i.e. demographic characteristics, but not the section where they were required to rate the importance of the topics. These respondents were not included in the analysis.

Overall, most respondents were health professionals (81%) and had between 5 and 20 years of experience in practice (63%) (Table 4). About a third of participants spent more than 75%, or between 50 and 75%, of their time in direct patient care. Most participants were primarily based at a teaching hospital (41%), and at a hospital (17%) or university (16%).

**Table 4:** Characteristics of online survey respondents

Characteristic	South Africa (*n* = 25)	Malawi (*n* = 20)	Nigeria (*n* = 53)	TOTAL (*n* = 98)
Primary stakeholder group	*n*			*n (%)*
Policymaker	5	2	1	8 (9)
Guideline developer	1	0	1	2 (2)
Health professional	16	13	47	76 (81)
Civil society representative	1	0	1	2 (2)
Researcher	1	2	2	5 (5)
Regional institution representative	0	1	0	1 (1)
Patient Advocacy representative	0	0	1	1 (1)
Other	1	2	0	3 (3)
Years of practice				
Less than 5	0	2	3	5 (5)
5 to 11	9	11	9	29 (31)
11 to 20	10	7	13	30 (32)
More than 20	6	0	28	34 (36)
Time spent in direct patient care				
Less than 25%	5	5	3	13 (14)
25 to 50%	2	7	11	20 (21)
51 to 75%	5	4	15	24 (26)
More than 75%	9	2	18	29 (31)
None	4	2	5	11 (12)
Primary institution				
Government	6	2	2	10 (11)
University	1	6	9	16 (17)
Hospital	6	4	5	15 (16)
NPO/NGO	3	4	3	10 (11)
Research	1	0	0	1 (1)
Teaching hospital	7	2	30	39 (41)
Private Sector			3	3 (3)
Other	1	0	1	2 (2)

NPO: Non-profit organization; NGO: non-governmental organization

In SA, nine of 14 topics were rated as *critically important*, five were rated as *very important*, and no topic was rated as *very critically important* (Tables 5, 6, 7). In Malawi, 10/30 topics were rated as *very critically important*, 14 as *critically important*, five as *very important*, and one as *important*. In Nigeria, 11/27 topics were rated as *very critically important*, 13 were rated as *critically important*, one as *very important*, one as *important* and one as *not important*. The topics that were taken through to the discussion with the Steering Groups included all the *critically important* topics in South Africa, and all the *very critically important* topics in Malawi and Nigeria. Individual ratings for each topic included in the survey of each country are presented in the Additional file 1: Fig. S1–S3.

**Table 5:** Overall rating of topics included in the survey in South Africa

	Topics	Median score*	IQR
	South Africa		
1	*Criteria for identifying children who require treatment for wasting in an outpatient/community setting*	5	1
2	*Technique for neonatal resuscitation*	5	1
3	*Ongoing care following discharge of premature baby*	5	1
4	*Developmental supportive care in hospital and after discharge for premature baby*	5	1
5	*Follow-up interventions for infants and children after discharge from treatment for wasting*	5	1.5
6	*Community level interventions to prevent stunting*	5	1.75
7	*Community level interventions to prevent mild, moderate and severe wasting*	5	2
8	*Neonatal resuscitation—role of oxygen, when to stop, care immediately post resuscitation*	5	2
9	*Management of meconium exposed baby*	5	3
10	Package of care for the management of children with cerebral palsy	4	1
11	Preventive chemotherapy to control schistosomiasis in endemic communities (mass drug administration)	4	1.5
12	Management of overweight and obesity in children	4	2
13	Prevention of overweight and obesity in children	4	2
14	Timing of cord clamping for normal or depressed newly-born babies	4	2.5

Italics emphasised topics were rated of highest importance and went through for discussion at SG meetings; *1: not important at all; 2: not important; 3: important; 4: very important; 5: critically important; 6: very critically important

**Table 6:** Overall rating of topics included in the survey in Malawi

	Topics	Median score*	IQR
	Malawi		
1	*Effectiveness of nutritional interventions (breastfeeding, Vitamin A supplementation) for under five children with TB and HIV who are malnourished*	6	1
2	*Effectiveness of community-based interventions (e.g., homebased care) to enhance adherence to ART in under-5 children with HIV/AIDS*	6	1
3	*Effectiveness of interventions for prevention of mother-to-child transmission of HIV*	6	1
4	*Interventions for in-patient management of children with severe acute malnutrition*	6	1.25
5	*Increasing effectiveness of implementation of WASH interventions (increasing boreholes or reliable water sources in low resource settings) among under 12 children in endemic areas*	6	1.25
6	*Mass immunization of under five children at all levels of care (i.e.at primary, secondary and tertiary level)*	6	1.5
7	*Accuracy of screening tests for suspected polio cases in under five children*	6	1.5
8	*Screening methods to use to identify HIV/AIDS children with nutritional deficiencies*	6	2
9	*Protocol (s) for under-five children with feeding difficulties (e.g., those with Cerebral Palsy, Cleft Palate and Hydrocephalus)*	6	2.5
10	*Effects of concurrent use of anti-TB drugs with first-line Antiretroviral therapy (ARTs) among children under the age of 12*	6	3
11	*Effectiveness of community-based interventions to prevent pneumonia in under-five children*	5.5	1.75
12	Effectiveness of training interventions (i.e., for new regimen and clinical management of HIV/AIDS) for health care providers caring for children with HIV/AIDS at primary level	5.5	2
13	Effectiveness of malaria school-based prevention and control interventions for children under 12 years of age	5.5	2
14	Accuracy of diagnostic tests for malaria in children under the age of 12 years	5.5	2.25
15	"Monitoring the effectiveness of mass vaccination campaigns to enhance Uptake of polio vaccination among under- five children	5.5	2.25
16	"Home management of mild/uncomplicated Falciparum malaria disease in children under-five years old	5.5	3
17	Effectiveness of cotrimoxazole for children (under the age of12) with co-morbid TB and HIV, with and without antiretroviral therapy	5.5	3
18	Early diagnosis of schistosomiasis among under-12 children in endemic areas	5.0	1
19	"Promotion of schistosomiasis Vector control of water sources (i.e., treatment of water sources, boiling of drinking water) among under 12 children in endemic areas"	5.0	1
20	Screening policies to improve identification of tuberculosis cases (including active case finding) in under 12 children	5.0	2
21	"Assessment of Post- treatment care (i.e., chest physiotherapy and nutritional support) for TB in under-five children"	5.0	2
22	"Interventions to reduce stigma and discrimination of children under 12 years old with HIV/AIDS"	5.0	2
23	"First-line treatment for non-severe pulmonary TB in under-five children"	5.0	3
24	Mobile technology app (mHealth) for management, reporting and monitoring acute malnutrition in under- 5 children	5.0	3
25	Social mobilization for health workers at all levels of care for schistosomiasis campaign in low-resource settings"	4.5	1.25
26	Effectiveness of Isoniazid treatment in children under 5 years old	4.5	2.5
27	Effectiveness of Praziquantel for treatment of schistosomiasis in under-five children"	4.0	0.5
28	Effectiveness of strategies to enhance uptake of vaccination in under -12 children"	4.0	2
29	Effectiveness of using Unisex Reference Chart to accommodate both male and female children	4.0	2.25
30	Effectiveness of Covid-19 vaccination with Pfizer for children < 12 years of age	3.0	1.5

Italics emphasised topics were rated of highest importance and went through for discussion at SG meetings

*1: not important at all; 2: not important; 3: important; 4: very important; 5: critically important; 6: very critically important

**Table 7:** Overall rating of topics included in the survey in Nigeria

	Topics	Median score*	IQR
	Nigeria		
1	*Interventions for improving identification and early referral of high-risk pregnancies*	6.0	0
2	*Management of infants with clinical severe infection or critical illness*	6.0	1
3	*Management of birth complications in preterm babies*	6.0	1
4	*Education of mothers on cord care for newborns*	6.0	1
5	*Interventions for improving birth preparedness*	6.0	1
6	*Health system interventions to improve access to vaccination for children in hard-to-reach areas*	6.0	1
7	*Interventions for promoting early breastfeeding in neonates and exclusive breastfeeding of infants*	6.0	1
8	*Education of mothers on thermal care for newborns*	6.0	1
9	*Educational Interventions to improve caregivers' treatment seeking behaviours for diarrhoea*	6.0	1
10	*Educational interventions to improve the uptake of childhood immunization*	6.0	1
11	*WASH interventions for preventing diarrhoea in under-five children*	5.5	2
12	Delamanid as long-dose regimens for treating children below 3 years with Multi-Drug Resistant//Rifampicin Resistant-TB	5.0	1
13	Interventions to improve the uptake of pneumococcal vaccines in children under five	5.0	1
14	Educational interventions for improving caregivers' treatment seeking behaviours for acute respiratory infections in children under five years of age	5.0	1
15	Home management of diarrhoea	5.0	1
16	Interventions to improve treatment coverage for severe acute malnutrition in children	5.0	1
17	Local nutritional formulations for under-fives with moderate acute malnutrition (MAM)	5.0	1
18	Interventions for improving TB case detection in children under five	5.0	1.5
19	Educational interventions for preschool and schoolteachers to improve immunization coverage	5.0	2
20	Involvement of Men in Postnatal Care and Maternal and Newborn Health	5.0	2
21	Antibiotics for prevention of neonatal sepsis or suspected neonatal sepsis	5.0	2
22	Community Health Workers Home visits for postnatal care to prevent neonatal sepsis	5.0	2
23	Prevention of Birth defects in the newborn	5.0	2
24	Prophylactic aminophylline for preventing apnea of prematurity	5.0	2
25	Identification and Management of Ophthalmia Neonatorum	4.1	1
26	Prevention of Trachoma in Children	4	2
27	Whole Body Massage for Growth and Development of Healthy Newborns	3	3

Italics emphasised topics were rated of highest importance and went through for discussion at SG meetings

*1: not important at all; 2: not important; 3: important; 4: very important; 5: critically important; 6: very critically important

Some survey respondents suggested additional topics. In South Africa 12/25 people suggested an additional 21 topics, six of 16 respondents in Malawi suggested an additional 15 topics, and 30 of 53 respondents in Nigeria suggested an additional 63 topics. In general, there was little overlap in the additional topics suggested and none were taken up as potential priorities across the countries.

#### Consensus meeting with Steering Groups

The main consensus meetings were held between November and December 2022 across the three countries. These were attended by members of the SG and research teams in each country. Additional individuals who joined the main Steering Group meetings included observers (*n* = 2), the Malawi and Nigeria project leads (*n* = 2) at the South African meeting, the project coordinator (TK) in Malawi, and a representative from the Department of Health Planning, Research and Statistics in Nigeria.

**Fig. 3:**
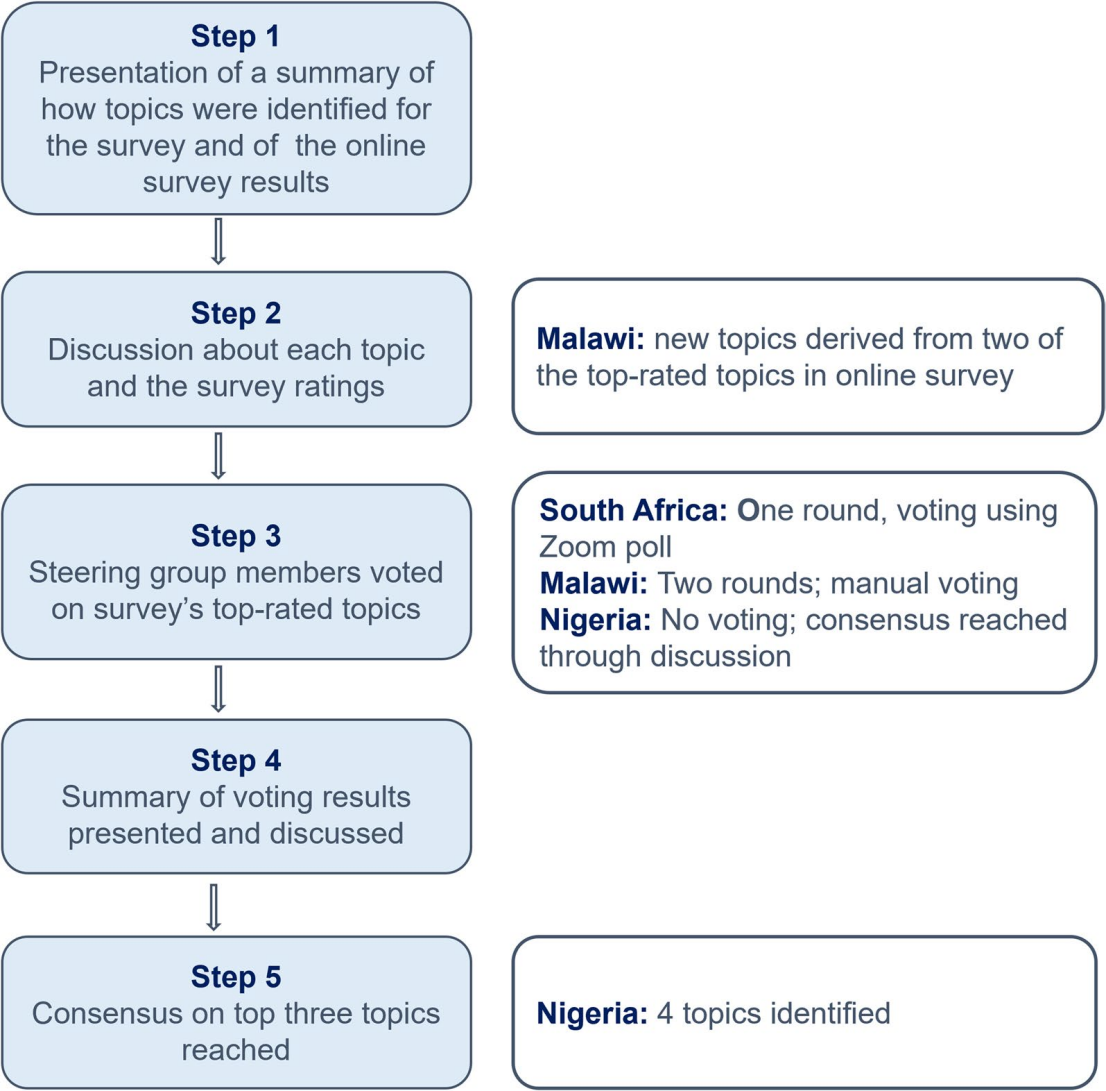
Flow diagram of the steps in the modified Nominal Group Technique

After the presentation of the results of the online survey (Step 1) and discussion about the top-rated topics (Step 2) (Table 5), facilitated by the moderator, the Steering Group members voted to identify the top three topics (Step 3). In South Africa, one round of voting indicated some consensus; of seven people who voted, three topics were rated as *critically* or *very critically important* by most people, and three topics were not rated by any member as *critical/very critical*. After further discussion on the results of the voting (Step 4), four topics were prioritized (Step 5). Figure 3 describes what took place in each step. In Malawi, although 10 top-rated topics were presented, the Steering Group members derived 10 new topics from topic 1 and 9, and these were the topics voted on. Two rounds of voting were done, after which three topics were identified. In Nigeria, the Steering Group agreed to reach decisions on topics by consensus. After extensive deliberations, considering the prevalence of health problems in neonates and the primary causes of these, they decided on four priority topics to consider.

Across all three countries, the topics selected by the end of the consensus meeting were very broad, i.e., each one encompassed many potential questions and was not yet sufficiently specific for a guideline process (i.e., in the Population, Intervention, Comparator, Outcomes—PICO format). Therefore, research teams had to do further work to unpack and refine these. To clarify the PICO questions linked to each topic, the teams compared existing national guidance on prioritised topics with recommendations in relevant WHO guidelines and other global guidance; this allowed identification of gaps in national guidance that the project could address. In Malawi, the team also consulted with experts in the field. This process resulted in seven potential PICO questions in South Africa, six in Malawi, and four in Nigeria. These were presented to the Steering Groups in additional meetings and via email communication, several rounds of which were required before final PICO questions were identified (Table 8). The final topics were also discussed with stakeholders responsible for developing and implementing national guidance, to clarify whether they linked to national priorities and whether they could fit within existing guideline development processes and infrastructure. Only topics that fit these conditions were taken through to the next stage of the project, the development of recommendations.

**Table 8:** Final priority PICO questions identified in the three countries

	PICO 1	PICO 2	PICO 3
South Africa	Iron supplementation in infants and children aged 6–23 months for preventing anaemia	Iron-containing micronutrient powders for point-of-use fortification of foods for infants and young children aged 6–23 months to prevent anaemia	Post-discharge preparation interventions for families with preterm and LBW infants
Malawi	Early versus delayed enteral nutritional interventions for reducing in-hospital morbidity and mortality in critically ill children aged 1 month to 12 years	Effective community-based interventions for improving early diagnosis of childhood cancers	Effective care interventions for neonates (new-born up to 28 days of age) for improving child mortality at primary care level
Nigeria	Interventions for identification and early management of pre-eclampsia in communities and primary health care facilities	Health worker-related Interventions to improve compliance with hand hygiene recommendations for infection prevention and control in hospitalized neonates and infants	Early versus delayed enteral feeding for improving outcomes in Low Birth Weight and Preterm Infants

## Discussion

We conducted a priority setting exercise to identify topics and inform new guideline development addressing gaps in newborn and child health in South Africa, Malawi, and Nigeria. In each country, the process included engagements with national Steering Groups comprising representatives of various national-level organisations, multi-stakeholder online surveys, and consensus meetings. At the end of the process three priority PICO questions were identified in each country. In South Africa, the topics concerned anemia prevention in infants and young children through iron supplementation and multiple micronutrient powders, and post-discharge support for caregivers of preterm and LBW babies. In Malawi, they focused on enteral nutrition in critically ill children, diagnosis of childhood cancers in the community, and caring for neonates. In Nigeria, the topics focused on identifying pre-eclampsia in the community, hand hygiene compliance to prevent infections, and enteral nutrition for LBW and preterm infants.

The topics identified are informing the next stages of the GELA project, which include a systematic guideline adaptation process, including scoping existing guidelines or systematic reviews addressing the topics, conducting evidence synthesis where necessary, and convening of guideline panels to make recommendations linked to some or all of the priority PICO questions in the three countries [10]. Through this process we are aiming to highlight best practice methods for guideline development, including priority setting with the involvement of relevant stakeholders through a transparent and systematic process, and through ensuring the guidelines are developed following rigorous methods and clear and transparent reporting.

The priorities identified at the end of the process had limited overlap across the three countries. This highlights the importance of contextualised priority setting processes, one of the good practice principles of priority setting [23]. Although contextualised priority setting is important because contextual factors drive the needs and the gaps in healthcare delivery and implementation in different countries, there can also be common priorities and issues. Therefore, countries with similar priorities could draw on existing work at a global or country level, for adaptation into their context, to prevent any regional system fragmentation.

Some of the topics included in the online survey were broad, which made it difficult to identify the questions in required format for a guideline question (PICO) at the end of the main expert consensus meetings. This required substantial work to refine the topics, as well as additional meetings with the Steering Groups, which delayed the finalisation of priority topics and the next steps of the project that depended on priority topics being identified. Ideally, the topics included in the survey should have been more specific. Otherwise, some of the work to clarify the top-rated topics identified through the survey could have been done before the Steering Group consensus meeting, to ensure better efficiency. Future priority setting should also consider more frequent meetings with national Steering Groups advising on topics, given the iterative nature of the process. The process may have been more efficient and easier if the starting point had been a narrower topic area, rather than covering all of newborn and child health.

Although this project aimed to identify priority topics in PICO format that would lead to one recommendation, this may not have been clear to all stakeholders involved. In some cases, stakeholders identified topics that were broad in nature and that would lend themselves for a full guideline encompassing different recommendation. This should be better clarified at the start of the process, when engaging with the stakeholders.

We noted better response rates to the online survey where emails inviting stakeholders to participate in the survey were from a recognised institutional address, and supplemented with WhatsApp communication, a method increasingly recognised as valuable for sharing digital health information [37].

In some cases, certain Steering Group members tended to dominate the consensus discussions. Management of stakeholder input during these meetings is a critical required skill for successfully gathering everyone’s views. In a study done to prioritise childhood cancer supportive care topics for the development of guidelines, Loeffen and colleagues chose to do a Delphi survey as one of the strengths of this method is the lack of face-to-face meetings to prevent dominant voices being introduced [38].

## Strengths and limitations

We followed a good practice method for priority setting including stakeholder engagement and using an explicit process [23]. We convened Steering Groups with representatives from relevant national decision-makers to advise on the project and topics, working in close collaboration with Departments of Health, aiming to ensure the project addresses national priorities that could fill a gap in national guidelines and guideline development processes. The online survey facilitated engagement with a broader range of stakeholders, to ensure broad representation of views and perspectives. Furthermore, the guideline development groups that would be identified in the next stage of the project to review the evidence and develop recommendations for each of the identified questions would ensure representation of key stakeholders. We also used specific criteria to rate the importance of topics, which were derived from the literature and which also received input to ensure they were understandable and relevant.

Our study had a few limitations. The response rates to surveys were poor, and could perhaps have been improved if they had remained open for longer. We sent reminder emails to those participants we had invited to complete the survey, but it was not possible to do this for others who may have received the link from others. We did not include patients or carers in the survey; research suggests that their perspectives may differ regarding what treatment decisions are important [39, 40]. We did, however, include civil society groups which provided perspectives that consider equity and patient and caregiver perspectives.

## Conclusions

Through an explicit process, including stakeholder engagement, reviewing of existing global guidelines and burden of disease, and online surveys we identified three priority questions each in South Africa, Malawi and Nigeria for guidelines addressing newborn and child health. We found that the process was not linear but rather iterative in nature, requiring several engagements with stakeholders to help finalise the topics, as well as managing the conflicting priorities of different groups of stakeholders. Our experience highlights the importance of contextualised priority setting, as shown by the limited overlap in topics prioritised across the three countries, as well as of the relationships with key decisionmakers, who help define the priorities.

### Supplementary Information

**Additional file1: Figure S1.** Rating of importance of survey topics in South Africa. **Figure S2.** Rating of importance of Malawi survey topics. **Figure S3.** Rating of importance of the topics included in the Nigeria survey.

## Data Availability

The datasets used and/or analysed during the current study are available from the corresponding author on reasonable request.

## Abbreviations

GELA: Global Evidence Local Adaptation
NGO: Non-governmental organisation
NPO: Non-profit organisation
