# Supplementary material for: Using a priority setting exercise to identify priorities for guidelines on newborn and child health in South Africa, Malawi, and Nigeria
Source: Health Res Policy Syst. 2024 Apr 16;22:48. doi: 10.1186/s12961-024-01133-7 (PMC11020907; doi:10.1186/s12961-024-01133-7)
Supplement: Supplementary file 1 — Additional file1: Figure S1. Rating of importance of survey topics in South Africa. Figure S2. Rating of importance of Malawi survey topics. Figure S3. Rating of importance of the topics included in the Nigeria survey. [file 12961_2024_1133_MOESM1_ESM.docx]

# Additional file

# 1. Individual ratings for each topic included in the survey of each country


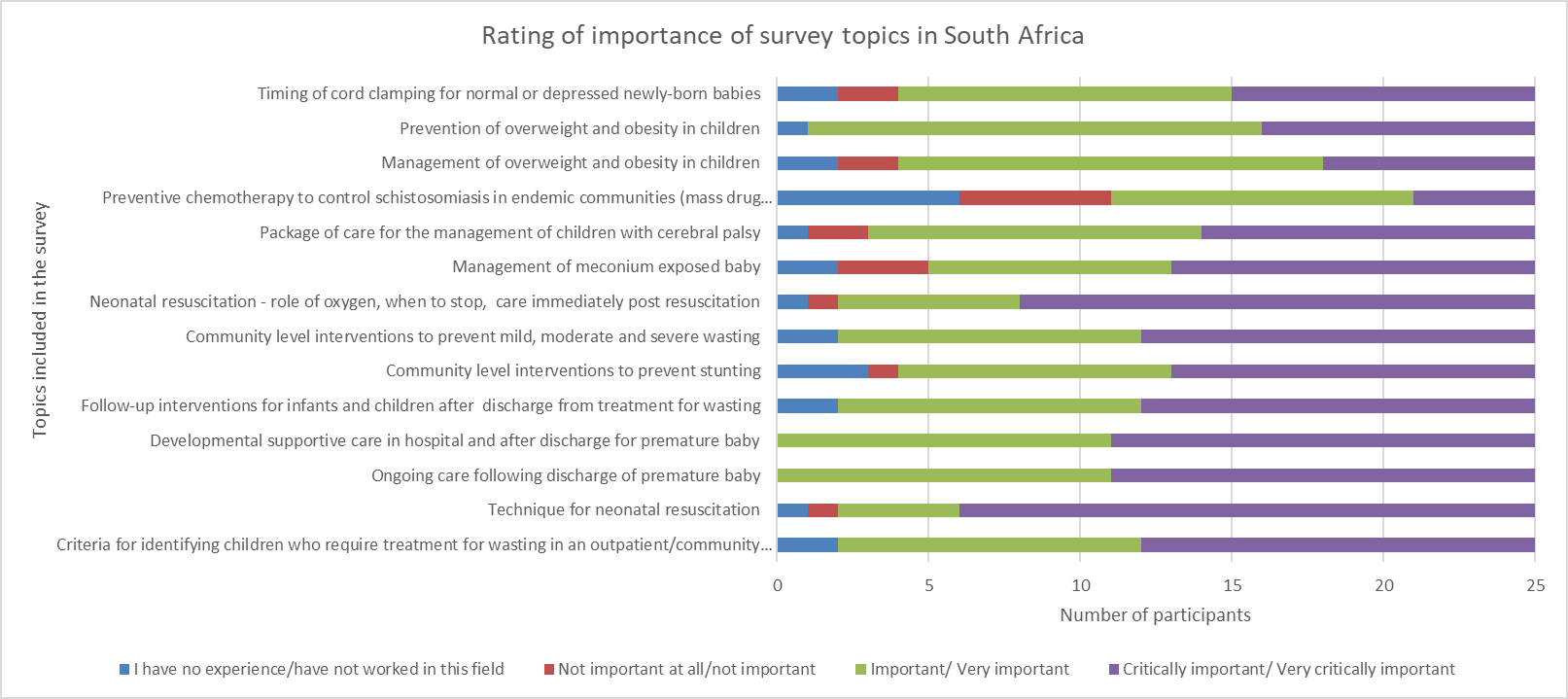


Figure S1. Rating of importance of survey topics in South Africa


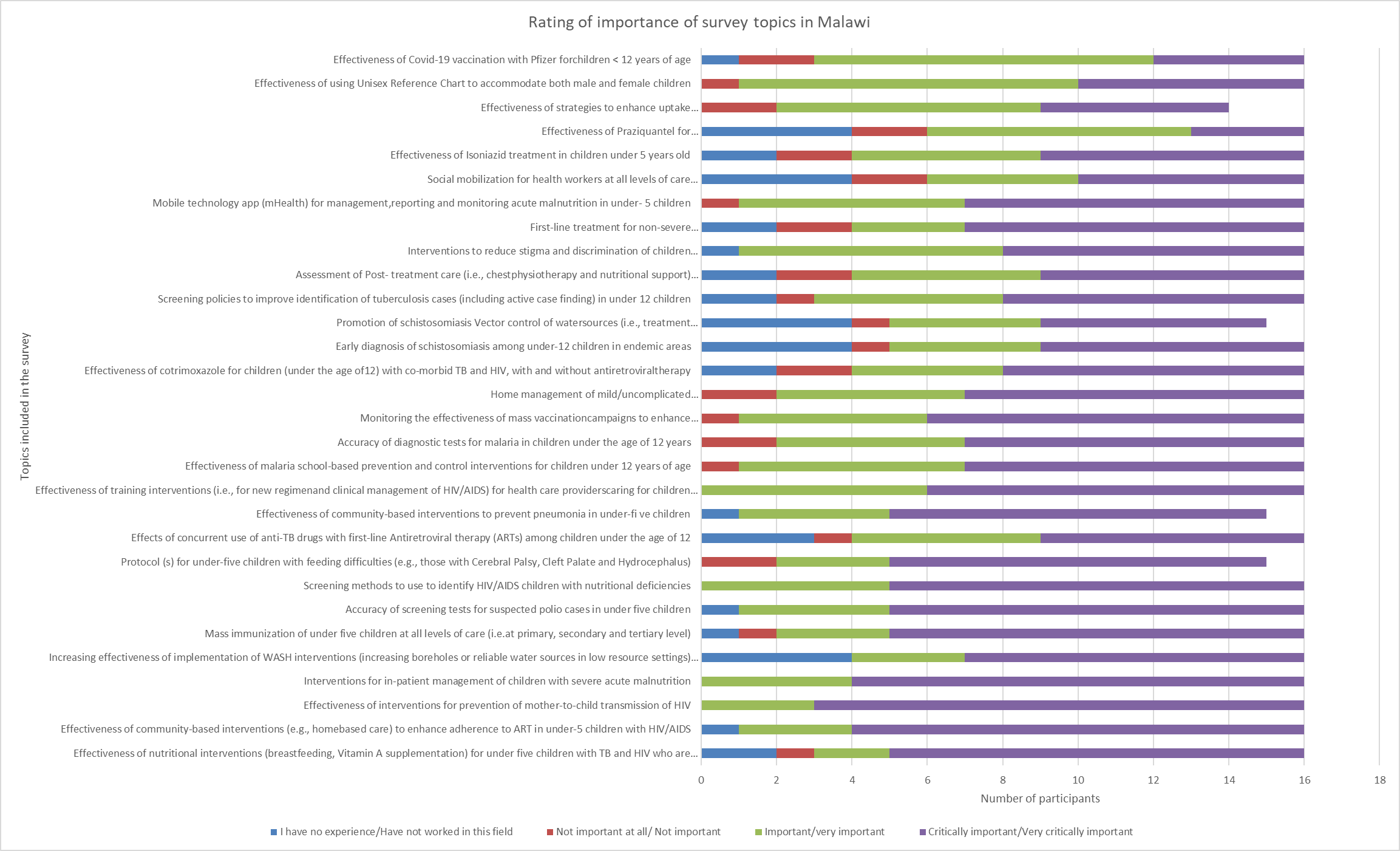


Figure S2. Rating of importance of Malawi survey topics


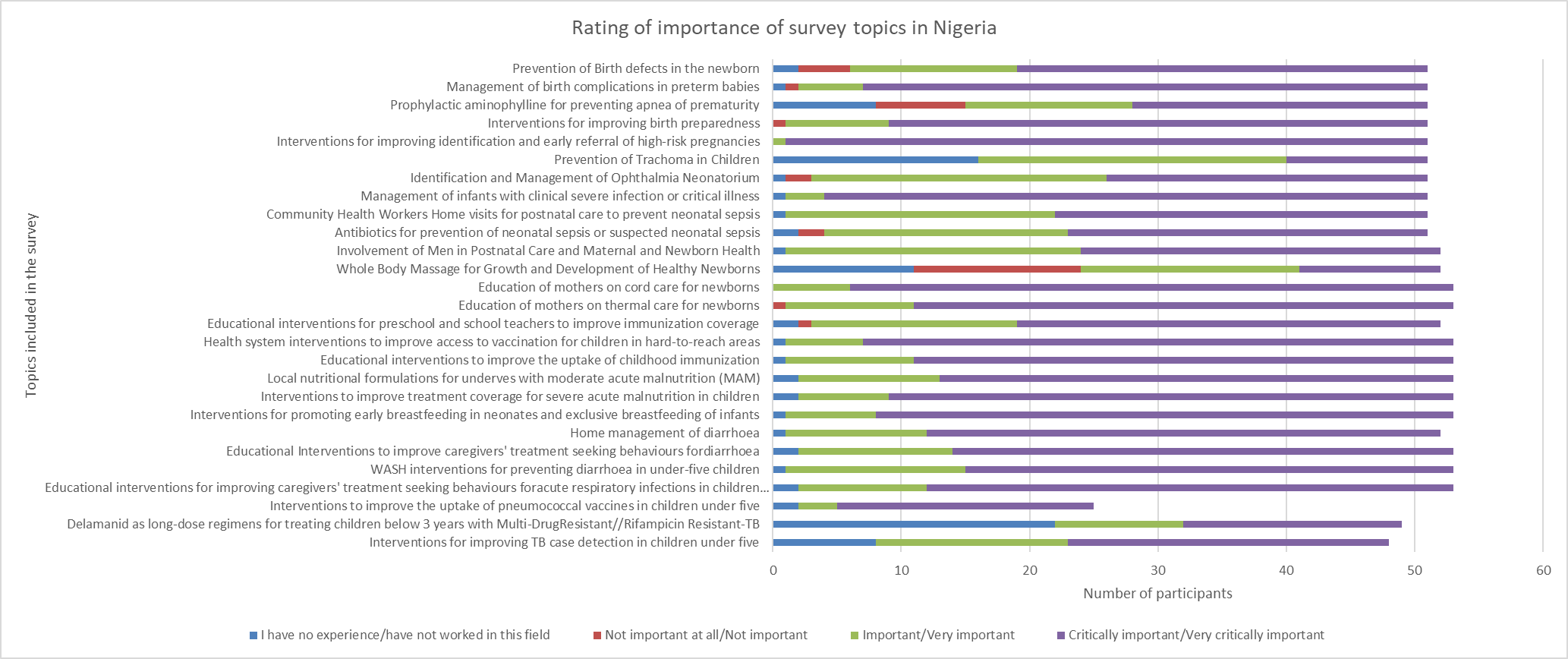


Figure S3. Rating of importance of the topics included in the Nigeria survey
